# Effectiveness of TNF inhibitors in patients with very early axial spondyloarthritis, defined as duration of ≤1 year of back pain: longitudinal observational data from the SCQM registry

**DOI:** 10.1136/rmdopen-2025-006647

**Published:** 2026-03-05

**Authors:** Mauro Bachmann, Andrea Götschi, Annik Steimer, Jonas Brändli, Kristina Bürki, Michael Andor, Claudia Lourenço Rodrigues, Simon Grosswiler, Martin Wendiggensen, Diego Kyburz, Michael J Nissen, Burkhard Möller, Sabine Adler, Diana Dan, Frauke Förger, Oliver Distler, Sofia Ramiro, Raphael Micheroli, Adrian Ciurea

**Affiliations:** 1 Department of Rheumatology, Zürich University Hospital, University of Zürich, Zürich, Switzerland; 2 Swiss Clinical Quality Management in Rheumatic Diseases Foundation, Zürich, Switzerland; 3 Rheumatologie im Zürcher Oberland, Uster, Switzerland; 4 Ankylosing Spondylitis Association, Zürich, Switzerland; 5 Johns Hopkins University School of Advanced International Studies, Washington, DC, USA; 6 Eidgenössische Technische Hochschule Zurich, Zürich, Switzerland; 7 Department of Rheumatology, University Hospital Basel, University of Basel, Basel, Switzerland; 8 Department of Rheumatology, Geneva University Hospital, Geneva, Switzerland; 9 Department of Rheumatology and Immunology, Inselspital, University Hospital Bern, University of Bern, Bern, Switzerland; 10 Department of Rheumatology, Kantonsspital Aarau, Aarau, Switzerland; 11 Department of Rheumatology, Lausanne University Hospital, University of Lausanne, Lausanne, Switzerland; 12 Department of Rheumatology, Kantonsspital St. Gallen, St. Gallen, Switzerland; 13 Department of Rheumatology, Leiden University Medical Center, Leiden, The Netherlands; 14 Department of Rheumatology, Zuyderland Medical Centre Heerlen, Heerlen, The Netherlands

**Keywords:** axial spondyloarthritis, biological therapy, tumor necrosis factor inhibitors

## Abstract

**Objective:**

To characterise patients with ‘very early’ axial spondyloarthritis (axSpA), defined as a duration ≤1 year of back pain and to determine the effectiveness of a first tumour necrosis factor inhibitor (TNFi) in very early versus established axSpA in a large observational registry.

**Methods:**

We included a total of 3324 patients with axSpA from the Swiss Clinical Quality Management in Rheumatic Diseases registry with available data on duration of back pain (≤1 year=very early axSpA, n=441; >1 year and ≤2 years=early axSpA, n=218; >2 years=established axSpA, n=2575). A first TNFi was started in 31%, 38% and 36% of patients with very early, early and established axSpA. Adjusted logistic regression models were used to compare the probability of achieving low disease activity status according to the Axial Spondyloarthritis Disease Activity Score (ASDAS <2.1) at 1 year. Drug survival was analysed with multiple-adjusted Cox proportional hazards models. Missing data were handled using multiple imputation by chained equations.

**Results:**

Objective signs of inflammation were more prevalent in very early disease. No difference was found regarding the achievement of ASDAS <2.1 after adjustment for age, sex, human leucocyte antigen-B27 status, education, body mass index, smoking, ASDAS and sacroiliac inflammation on MRI (OR 1.08, 95% CI 0.70 to 1.68 in very early vs established axSpA). Similarly, no significant difference in TNFi retention was found in very early versus established axSpA (HR for drug discontinuation 1.05, 95% CI 0.84 to 1.31).

**Conclusion:**

We found no evidence for a better effectiveness of TNFi in patients with very early versus established axSpA.

**WHAT IS ALREADY KNOWN ON THIS TOPIC**

- According to a consensus by the Assessment in SpondyloArthritis international Society, early axial spondyloarthritis (axSpA) is defined as having ≤2 years of axial symptom duration.
- Initiation of a biological or targeted-synthetic disease-modifying antirheumatic drug during this early disease phase was not found to result in better short-term treatment effectiveness compared with later initiation in recent studies.
- Whether starting treatment within an even shorter symptom duration threshold (≤1 year) leads to better outcomes remains unknown.

**WHAT THIS STUDY ADDS**

- This study characterises an axSpA population with very early disease, defined as ≤1 year of back pain.
- No evidence was found for a better effectiveness of tumour necrosis factor inhibitors in patients with very early axSpA compared with established axSpA in terms of 1-year clinical outcomes and drug survival.

**HOW THIS STUDY MIGHT AFFECT RESEARCH, PRACTICE OR POLICY**

- Further analyses are needed to investigate the long-term benefits of an early intervention, particularly regarding potential reductions in structural damage.

## Introduction

 Whether a ‘window of opportunity’ exists in axial spondyloarthritis (axSpA) remains a subject of debate.^1 2^ This concept proposes that initiating treatment early may lead to improved clinical outcomes and potentially reduce long-term structural damage. However, since various definitions of ‘early disease’ have been used in previous clinical trials in axSpA,^3^ the members of the Assessment of SpondyloArthritis international Society (ASAS) have reached a consensus to define ‘early axSpA’ as a duration of ≤2 years of axial symptoms.^4^ This definition was considered optimistic, as the current global mean diagnostic delay for axSpA remains substantial (5.6 years).^5^

The new ASAS definition for early axSpA has already been used to assess the short-term clinical outcome in a few studies. In a Swiss observational study, the initiation of a first tumour necrosis factor inhibitor (TNFi) during the early axSpA phase was not found to be associated with better drug retention or higher response rates compared with starting treatment later.^6^ Comparable response rates in both early and established disease were also observed in post hoc analyses of randomised controlled trials of upadacitinib and bimekizumab in axSpA.^7 8^ Furthermore, a meta-analysis of 11 trials found no significant differences in the efficacy of biological disease-modifying antirheumatic drugs (bDMARDs) across thresholds ranging from 2 to 5 years of symptom duration.^9^ A shorter cut-off for early disease could not be analysed in this meta-analysis due to the low number of patients who started a bDMARD within 1 year of axial symptom onset in the majority of axSpA trials included.

Given the lack of available data on treatment outcomes in early disease using even shorter symptom duration thresholds, we thought to analyse the effectiveness of a first TNFi in patients with ‘very early axSpA’, defined as a duration ≤1 year of back pain, compared with those with longer symptom duration, using the Swiss Clinical Quality Management in Rheumatic Diseases (SCQM) patient registry.^10^

## Methods

### Study population

Patients diagnosed with axSpA by their treating rheumatologist and registered in the SCQM axSpA cohort^9^ between 1 January 2004 and 1 April 2025 were included in this study, provided that information on the onset of axial symptoms was available. The treating rheumatologists record the date of the first symptoms in the online database on the patient’s inclusion in the SCQM registry. They are then prompted to indicate whether the patient has experienced back pain lasting ≥3 months (yes or no) based on the interpretation of the patient’s medical history. If the answer is ‘yes’, the rheumatologist is asked to select various items from a checklist to assess whether the back pain meets the criteria for inflammatory back pain according to ASAS, followed by the starting date of back pain. The onset date of axial symptoms, as recorded in the SCQM registry, refers solely to back pain (not morning stiffness).^6^ Assessments of patients at inclusion and subsequent visits are performed in accordance with ASAS recommendations.^11^ C reactive protein (CRP) levels were collected together with information on the threshold at which the respective laboratory considered the value to be elevated.

Disease stages were determined at two time points: (1) at inclusion in the SCQM registry for comparison of patient characteristics and (2) at initiation of first TNFi therapy for analyses of treatment effectiveness. At each time point, patients were categorised as having either (a) very early disease (≤1 year of back pain duration at the corresponding reference date), (b) early disease (duration of back pain >1 year and ≤2 years) or (c) established disease (>2 years of back pain duration). Onset dates recorded as 1 January or 1 June were interpreted as approximate placeholders indicating that the exact onset date was unknown. These cases were therefore treated as interval-censored, assuming symptom onset occurred at an unspecified time within that calendar year. Patients for whom disease stage could not be determined unambiguously—because the 1-year threshold overlapped with the reference date—were excluded from the analysis.

### Effectiveness of treatment with a first TNF inhibitor in very early versus established axSpA and early versus established axSpA

Data on the use of TNFi are entered by the rheumatologist with start and discontinuation dates. In a first exploratory analysis, we analysed changes in CRP levels over time in patients with very early, early and established axSpA using a log-linear regression model. CRP values were log-transformed prior to the analysis to account for their skewed distribution. This approach estimates the relative difference in CRP at 1 year (±6 months) between the groups while adjusting for baseline CRP.

The primary outcome of our analysis of treatment effectiveness was the achievement of at least a low disease activity status (Axial Spondyloarthritis Disease Activity Score (ASDAS) <2.1) at 1 year (±6 months) after treatment initiation. Patients who had discontinued their first TNFi before this time point for reasons other than remission were considered non-responders (response/tolerance analysis).^12^ Additional response criteria assessed included achievement of remission (ASDAS inactive disease status corresponding to ASDAS <1.3), achievement of a clinically important improvement in ASDAS (ASDAS-CII) and achievement of a major improvement in ASDAS (ASDAS-MI). Drug survival was chosen as a secondary outcome. The hazard of treatment discontinuation was estimated for patients in the three groups with established axSpA serving as reference group. Observations were censored either at the last visit in the SCQM registry or the patient’s last confirmation of the use of the respective TNFi agent via the mySCQM application.

### Statistical analyses

R statistical software (V.4.4.2) was used for all statistical analyses.^13^ Baseline characteristics of patients with very early, early and established axSpA were compared using the Kruskal-Wallis rank-sum test for continuous variables and the χ^2^ test for categorical variables. All tests were two-sided, with a significance level of 0.05.

Logistic regression was applied to estimate unadjusted and adjusted ORs for treatment response in patients with very early versus established disease and in patients with early disease versus established disease. Model 1 was adjusted for potential confounders such as age, sex (female vs male), human leucocyte antigen-B27 (HLA-B27) status (negative vs positive) and education (vocational vs compulsory and academic vs compulsory). Model 2 included additional adjustments for ASDAS, inflammatory changes on MRI of the sacroiliac joints (yes vs no), body mass index (BMI) and smoking status (current vs former/never) all at baseline. Crude time to discontinuation of the first TNFi was compared between groups with log-rank tests. Multiple adjusted Cox proportional hazards models were used to estimate HRs for drug discontinuation in patients with very early axSpA versus established axSpA and in early axSpA versus established axSpA. The adjusted models included the same covariates as the response analyses. As drug survival might also depend on the number of therapeutic options, we conducted a sensitivity analysis by adding a time-period variable to model 2 (start of treatment 2015–2025 vs 2004–2014 as the reference).

We anticipated that proportions of patients starting TNFi in the different symptom duration groups might differ across distinct time periods following the availability of additional therapeutic options after 2015; therefore, we conducted an additional sensitivity analysis.

Missing data were handled under the missing-at-random assumption using multiple imputation by chained equations (MICE package V.3.17.0). Sensitivity analyses were performed on patients without missing data (complete case analyses). In the imputation model for the response analyses, all covariates and all components of the outcomes were included.^14^ For the retention analyses, covariates were imputed based on all covariates, the time-to-event variable transformed using the cumulative hazard function and the event indicator. For derived variables (eg, BMI, ASDAS), only their components were included in the models. Composite variables (ASDAS improvements), BMI and elevated CRP were passively imputed. Auxiliary variables (Physician Global Assessment, Bath Ankylosing Spondylitis Disease Activity Index (BASDAI) at treatment start and follow-up for the response analyses, swollen joint count 28 and time from back pain onset to diagnosis) were incorporated in both models to improve imputation quality.

Based on the maximum number of patients with at least one missing value, we generated 65 imputations with 50 iterations, using predictive mean matching (five donors) for continuous variables, logistic regression for binary variables and proportional-odds models for ordered variables. Imputation quality was assessed using trace plots and by comparing the distributions of observed and imputed values. Sensitivity analyses using alternative imputation specifications yielded similar results. Estimates were pooled using Rubin’s rules.^15^

## Results

### Characterisation of patients with very early axSpA at inclusion in the SCQM registry

A total of 3234 patients with axSpA had available information on the date of onset of back pain, out of 5609 patients with a confirmed diagnosis of axSpA during follow-up (online supplemental figure S1). According to our definition of ‘very early’ axSpA (≤1 year of back pain duration), 441 patients met this criterion. An additional 218 patients were classified as having early axSpA (>1 year and ≤2 years of back pain) and 2575 as having established disease (>2 years of back pain). The proportion of patients with very early axSpA remained stable across calendar periods: 11% in 2011–2015, 11% in 2016–2020, 10% in 2021–2025. Demographic and clinical characteristics of the three groups are presented in table 1. The median (IQR) duration of back pain was 0.4 (0.1; 0.7) years in the very early axSpA group, 1.4 (1.2; 1.7) years in the early axSpA group and 10.1 (5.4; 18.8) years in the established axSpA group. Patients with very early axSpA were more frequently male and HLA-B27 positive. Objective signs of inflammation, such as elevated CRP and sacroiliac inflammation on MRI, were also more common in this group. No significant differences were observed between groups in the BASDAI and the Bath Ankylosing Spondylitis Functional Index. As expected, reflecting their substantially longer symptom duration, patients with established axSpA showed more impairment of spinal mobility, as assessed by the Bath Ankylosing Spondylitis Mobility Index. A higher proportion of patients with established axSpA was already receiving bDMARD therapy at inclusion in the SCQM registry.

**Table 1:** Characteristics of patients with axSpA with very early disease, early disease and established disease at inclusion in the SCQM registry

Parameter	N	Very early axSpA (≤1 year) N=441	N	Early axSpA (>1 and ≤2 years) N=218	N	Established axSpA (>2 years) N=2575	P value
Male sex, n (%)	441	244 (55)	218	97 (46)	2575	1333 (52)	0.03
Age, years	441	37.5 (12.6)	218	38.0 (12.5)	2575	43.3 (12.7)	<0.001
Symptom duration, years, median (IQR)	434	0.8 (0.6; 5.0)	216	1.5 (1.2; 1.8)	2551	10.5 (5.6; 19.2)	<0.001
Axial symptoms duration, years, median (IQR)	441	0.4 (0.1; 0.7)	218	1.4 (1.2; 1.7)	2575	10.1 (5.4; 18.8)	<0.001
Time since diagnosis, years, median (IQR)	434	0.3 (0.1; 0.6)	218	0.5 (0.2; 1.0)	2544	3.0 (0.6; 8.6)	<0.001
HLA-B27, n (%)	380	253 (67)	200	117 (54)	2302	1386 (54)	0.001
Family history of axSpA, n (%)	395	83 (21)	191	30 (16)	2269	529 (23)	0.04
Body mass index	392	24.7 (4.3)	190	25.9 (4.8)	2230	25.8 (4.7)	<0.001
Education	358		166		1939		0.02
Compulsory		47 (13)		18 (11)		347 (18)	
Vocational		211 (59)		97 (52)		1002 (52)	
Academic		100 (28)		51 (31)		590 (30)	
Recruiting rheumatologist	441		218		2575		<0.001
Private practice		252 (57)		117 (54)		1386 (54)	
Non-academic hospital		120 (27)		61 (28)		548 (21)	
Academic hospital		69 (16)		40 (18)		641 (25)	
ASAS 2009 classification criteria, n (%)	367	287 (78)	177	130 (73)	1922	1649 (86)	<0.001
Radiographic axSpA, n (%)	181	113 (62)	64	27 (42)	865	661 (76)	<0.001
IBP (ASAS definition) (ever), n (%)	397	245 (62)	187	120 (64)	2123	1637 (77)	<0.001
Sacroiliitis on MRI (inflammation)	434	238 (55)	211	133 (63)	2462	1044 (42)	<0.001
Peripheral arthritis (ever), n (%)	441	188 (43)	214	69 (32)	2527	1097 (43)	0.01
Enthesitis (ever), n (%)	441	302 (69)	217	128 (59)	2550	1674 (66)	0.054
Uveitis (ever), n (%)	341	41 (12)	163	8 (5)	1943	344 (18)	<0.001
Psoriasis (ever), n (%)	343	30 (9)	167	16 (10)	1973	106 (10)	0.61
Inflammatory bowel disease (ever), n (%)	327	33 (10)	160	12 (8)	1918	184 (10)	0.64
Fibromyalgia, n (%)	215	1 (1)	97	2 (2)	1573	50 (4)	0.02
Depression and/or anxiety, n (%)	245	11 (5)	127	14 (11)	1953	210 (11)	0.003
Physician global disease activity	425	4.1 (2.3)	212	3.4 (2.2)	2431	3.4 (2.2)	<0.001
Patient global disease activity	358	5.0 (2.8)	177	4.9 (2.7)	2038	4.8 (2.8)	0.38
BASDAI	344	4.6 (2.2)	163	4.3 (2.2)	1915	4.5 (2.3)	0.33
ASDAS	311	2.9 (1.0)	149	2.6 (1.0)	1693	2.7 (1.0)	0.003
Elevated CRP*, n (%)	403	169 (42)	199	46 (23)	2265	665 (29)	<0.001
CRP (mg/L), median (IQR)	406	5.9 (2.0; 12.7)	201	3.0 (1.0; 7.2)	2267	3.1 (1.0; 8.0)	<0.001
BASFI	345	3.0 (2.5)	162	2.5 (2.3)	1896	2.9 (2.5)	0.09
BASMI	406	1.5 (1.5)	191	1.2 (1.2)	2240	1.9 (1.8)	<0.001
SF-12, physical component summary score	326	38.0 (10.4)	155	40.5 (10.5)	1797	39.5 (10.3)	0.02
SF-12, mental component summary score	326	43.8 (11.1)	155	44.6 (10.6)	1797	43.7 (11.4)	0.77
Non-steroidal anti-inflammatory drugs, n (%)	400	371 (93)	194	174 (90)	2248	1921 (86)	<0.001
Conventional synthetic DMARDs, n (%)	441	57 (13)	218	24 (11)	2573	297 (12)	0.08
Tumour necrosis factor inhibitors, n (%)	441	134 (30)	218	98 (45)	2575	1254 (49)	<0.001
Interleukin-17 inhibitors, n (%)	441	4 (1)	218	4 (2)	2575	68 (3)	0.07

Except where indicated otherwise, values represent the mean and SD.

* CRP levels are collected together with information on the threshold at which the respective laboratory considers the value to be elevated.

ASAS, Assessment of SpondyloArthritis international Society; ASDAS, Axial Spondyloarthritis Disease Activity Score; axSpA, axial spondyloarthritis; BASDAI, Bath Ankylosing Spondylitis Disease Activity Index; BASFI, Bath Ankylosing Spondylitis Functional Index; BASMI, Bath Ankylosing Spondylitis Metrology Index; CRP, C reactive protein; DMARD, disease-modifying antirheumatic drug; HLA-B27, human leucocyte antigen-B27; IBP, Inflammatory back pain; SCQM, Swiss Clinical Quality Management in Rheumatic Diseases; SF-12, Short Form questionnaire with 12 questions.

### Characterisation of patients starting a first TNFi after inclusion in the SCQM registry

A total of 138 patients with very early axSpA, 83 patients with early axSpA and 928 patients with established axSpA initiated treatment with a first TNFi after being included in the SCQM cohort (online supplemental figure S1). The demographic and clinical characteristics of these patients are shown in table 2. Since these patients were bio-naïve, a higher proportion exhibited objective signs of inflammation, such as elevated CRP levels and active sacroiliitis on MRI, along with a higher BASDAI and ASDAS. With the exception of a higher prevalence of uveitis among patients with established axSpA, other extra-musculoskeletal and musculoskeletal manifestations were similarly distributed across the three groups. Some differences were observed between the groups regarding the proportion of patients starting TNFi between 2004 and 2014 and between 2015 and 2025 (table 2).

**Table 2:** Characteristics of patients with axSpA with very early disease, early disease and established disease at the start of the first TNF inhibitor

Parameter	N	Very early axSpA (≤1 year) N=138	N	Early axSpA (>1 and ≤2 years) N=83	N	Established axSpA (>2 years) N=928	P value
Male sex, n (%)	138	76 (55)	83	38 (46)	928	485 (52)	0.40
Age, years	138	37.9 (13.1)	83	40.3 (13.1)	928	43.4 (12.3)	<0.001
Symptom duration, years, median (IQR)	138	0.9 (0.6; 5.6)	81	1.4 (1.3; 1.8)	921	10.9 (5.9; 19.7)	<0.001
Axial symptoms duration, years, median (IQR)	138	0.5 (0.3; 0.7)	83	1.4 (1.1; 1.6)	928	10.2 (5.3; 18.9)	<0.001
HLA-B27, n (%)	123	71 (58)	74	39 (53)	855	569 (67)	0.01
Body mass index	127	24.5 (4.2)	73	25.5 (4.9)	854	25.8 (4.6)	0.01
Education	114		65		751		0.04
Compulsory, n (%)		17 (15)		4 (6)		127 (17)	
Vocational, n (%)		75 (66)		44 (68)		415 (55)	
Academic, n (%)		22 (19)		17 (26)		209 (28)	
ASAS 2009 classification criteria, n (%)	116	87 (75)	65	48 (74)	711	622 (88)	<0.001
Radiographic axSpA, n (%)	54	34 (63)	28	10 (36)	364	281 (77)	<0.001
IBP (ASAS definition) (ever), n (%)	122	79 (65)	70	46 (66)	718	579 (81)	<0.001
Sacroiliitis on MRI (inflammation)	130	75 (58)	72	45 (63)	803	365 (46)	0.001
Peripheral arthritis (ever), n (%)	134	65 (49)	77	31 (40)	856	364 (43)	0.37
Enthesitis (ever), n (%)	234	91 (68)	77	49 (64)	844	589 (70)	0.51
Uveitis (ever), n (%)	103	6 (6)	62	6 (10)	656	107 (16)	0.01
Psoriasis (ever), n (%)	103	12 (12)	66	8 (12)	668	76 (11)	0.98
Inflammatory bowel disease (ever), n (%)	101	8 (8)	60	5 (8)	656	54 (8)	0.99
Fibromyalgia, n (%)	66	0 (0)	37	0 (0)	597	24 (4)	0.07
Depression and/or anxiety, n (%)	80	3 (4)	53	4 (8)	725	65 (9)	0.19
Physician global disease activity	123	5.3 (1.9)	67	4.6 (2.0)	763	4.7 (1.8)	0.01
Patient global disease activity	106	6.4 (2.3)	63	5.6 (2.1)	670	6.0 (2.5)	0.07
BASDAI	105	5.7 (1.9)	56	5.1 (1.9)	653	5.3 (2.0)	0.04
ASDAS	96	3.4 (0.9)	48	3.0 (0.9)	592	3.2 (0.9)	0.01
Elevated CRP*, n (%)	119	60 (50)	66	27 (41)	734	328 (45)	0.39
CRP (mg/L), median (IQR)	120	6.8 (3.0; 16.0)	66	4.7 (1.1; 9.9)	735	6.0 (2.0; 13.0)	0.04
BASFI	104	3.9 (2.4)	54	3.1 (2.0)	632	3.6 (2.4)	0.15
BASMI	113	1.5 (1.4)	58	1.1 (1.0)	684	2.1 (1.9)	<0.001
SF-12, physical component summary score	95	34.9 (9.3)	48	37.8 (9.1)	572	36.3 (9.1)	0.13
SF-12, mental component summary score	95	40.5 (10.0)	48	42.4 (9.5)	572	42.2 (11.2)	0.37
Non-steroidal anti-inflammatory drugs, n (%)	90	87 (97)	45	43 (96)	537	509 (95)	0.74
Conventional synthetic DMARDs, n (%)	138	21 (15)	83	11 (13)	927	115 (12)	0.65
Start period of first TNFi	138		83		928		0.02
2004–2014		90 (65)		39 (47)		510 (55)	
2015–2025		48 (35)		44 (53)		418 (45)	

Except where indicated otherwise, values represent the mean and SD.

* CRP levels are collected together with information on the threshold at which the respective laboratory considers the value to be elevated.

ASAS, Assessment of SpondyloArthritis international Society; ASDAS, Axial Spondyloarthritis Disease Activity Score; axSpA, axial spondyloarthritis; BASDAI, Bath Ankylosing Spondylitis Disease Activity Index; BASFI, Bath Ankylosing Spondylitis Functional Index; BASMI, Bath Ankylosing Spondylitis Metrology Index; CRP, C reactive protein; DMARD, disease-modifying antirheumatic drug; HLA-B27, human leucocyte antigen-B27; IBP, Inflammatory back pain; SF-12, Short Form questionnaire with 12 questions; TNFi, tumour necrosis factor inhibitor.

### Treatment response analyses

Slightly higher crude response rates were observed in patients with very early axSpA compared with those with established axSpA after 1 year of treatment for the majority of analysed outcomes (table 3): 45% vs 42% for ASDAS <2.1 (OR 1.12, 95% CI 0.70 to 1.78), 20% vs 20% for ASDAS <1.3 (OR 0.99, 95% CI 0.54 to 1.73), 50% vs 40% for ASDAS-CII (OR 1.48, 95% CI 0.87 to 2.49) and 27% vs 18% for ASDAS-MI (OR 1.69, 95% CI 0.91 to 3.04). Statistical models, adjusted for potential confounders and disease activity variables after multiple imputation of missing covariates, are presented in tables4  5. The OR for achieving an ASDAS <2.1 in very early versus established axSpA was 1.06 (95% CI 0.69 to 1.63) after adjustment for age, sex, HLA-B27 and education (table 4). The OR remained similar after adjustment for baseline ASDAS, BMI, smoking and sacroiliac inflammation (OR 1.08, 95% CI 0.70 to 1.68; table 5). Additional adjustment for the time period of initiation of the first TNFi (from 2004 to 2014 vs 2015 to 2025) only marginally affected the estimates in our model (online supplemental table S2). A comparison between the results of this model using multiple imputation and a complete case analysis is shown in the online supplemental figure S2. The response rates for additional response outcomes (ASDAS <1.3, ASDAS-CII and ASDAS-MI) were also comparable between patients with very early and established disease and likewise between patients with early and established disease in these analyses (tables3^5^). Male sex, HLA-B27 positivity and higher baseline ASDAS levels were consistently associated with significantly better response rates across nearly all outcomes (tables4  5). Higher educational levels and lower BMI levels were additionally associated with better treatment responses in the models that included adjustment for baseline ASDAS (table 5).

**Table 3:** Unadjusted response rate analyses at 1 year of treatment with a first TNFi for different outcomes in very early, early and established axSpA

Outcome	N	Very early axSpA Events (%)	Early axSpA Events (%)	Established axSpA Events (%)	Comparison	OR	95% CI	P value
ASDAS <2.1	648	38 (45)	22 (46)	219 (42)	Very early versus established axSpA	1.12	0.70 to 1.78	0.63
					Early versus established axSpA	1.15	0.63 to 2.08	0.65
ASDAS <1.3	648	17 (20)	8 (17)	105 (20)	Very early versus established axSpA	0.99	0.54 to 1.73	0.98
					Early versus established axSpA	0.78	0.33 to 1.64	0.54
ASDAS-CII	488	33 (50)	12 (39)	158 (40)	Very early versus established axSpA	1.48	0.87 to 2.49	0.15
					Early versus established axSpA	0.93	0.43 to 1.95	0.85
ASDAS-MI	488	18 (27)	6 (19)	71 (18)	Very early versus established axSpA	1.69	0.91 to 3.04	0.09
					Early versus established axSpA	1.08	0.39 to 2.57	0.87

ASDAS, Axial Spondyloarthritis Disease Activity Score; ASDAS-CII, clinically important improvement in ASDAS; ASDAS-MI, major improvement in ASDAS; axSpA, axial spondyloarthritis; TNFi, tumour necrosis factor inhibitor.

**Table 4:** Multiple adjusted response rate analyses at 1 year of treatment with a first TNFi for different outcomes in very early and early versus established axSpA (model 1)

Model 1	ASDAS <2.1			ASDAS <1.3			ASDAS-CII			ASDAS-MI		
Variable	OR	95% CI	P value	OR	95% CI	P value	OR	95% CI	P value	OR	95% CI	P value
Very early versus established disease	1.06	0.69 to 1.63	0.79	0.97	0.56 to 1.70	0.92	1.30	0.84 to 2.00	0.24	1.47	0.86 to 2.50	0.15
Early versus established disease	1.10	0.63 to 1.93	0.73	0.78	0.37 to 1.66	0.53	0.98	0.56 to 1.73	0.95	0.99	0.47 to 2.08	0.98
Age	0.98	0.97 to 0.99	0.003	0.98	0.96 to 0.99	0.003	0.99	0.98 to 1.00	0.052	0.99	0.97 to 1.01	0.18
Female sex	0.44	0.33 to 0.59	<0.001	0.44	0.30 to 0.66	<0.001	0.49	0.36 to 0.66	<0.001	0.46	0.30 to 0.70	<0.001
HLA-B27 negativity	0.39	0.28 to 0.53	<0.001	0.41	0.25 to 0.68	<0.001	0.40	0.28 to 0.58	<0.001	0.41	0.25 to 0.68	<0.001
Education vocational versus compulsory	2.07	1.27 to 3.39	0.004	2.00	1.01 to 3.97	0.047	1.17	0.74 to 1.85	0.51	0.92	0.51 to 1.68	0.79
Education academic versus compulsory	3.18	1.89 to 5.35	<0.001	2.82	1.39 to 5.72	0.004	1.28	0.75 to 2.15	0.36	1.09	0.58 to 2.04	0.78

Response rates in patients with available outcome at 1 year (±6 months), patients having discontinued the biologic in the meantime for reasons other than remission being considered non-responders.

ASAS, Assessment in SpondyloArthritis international Society; ASDAS, Axial Spondyloarthritis Disease Activity Score; ASDAS-CII, clinically important improvement in ASDAS; ASDAS-MI, major improvement in ASDAS; axSpA, axial spondyloarthritis; HLA-B27, human leucocyte antigen-B27; TNFi, tumour necrosis factor inhibitor.

**Table 5:** Multiple adjusted response rate analyses at 1 year of treatment with a first TNFi for different outcomes in very early and early versus established axSpA after adjustment for additional potential confounders (model 2)

Model 2	ASDAS <2.1			ASDAS <1.3			ASDAS-CII			ASDAS-MI		
Variable	OR	95% CI	P value	OR	95% CI	P value	OR	95% CI	P value	OR	95% CI	P value
Very early versus established disease	1.08	0.70 to 1.68	0.73	0.85	0.48 to 1.52	0.59	0.99	0.62 to 1.58	0.96	0.96	0.51 to 1.81	0.89
Early versus established disease	1.04	0.59 to 1.84	0.90	0.73	0.34 to 1.56	0.41	1.10	0.59 to 2.04	0.76	1.17	0.50 to 2.76	0.71
Age	0.99	0.97 to 1.00	0.03	0.99	0.97 to 1.00	0.06	0.99	0.97 to 1.00	0.10	0.99	0.97 to 1.01	0.32
Female sex	0.41	0.30 to 0.55	<0.001	0.40	0.26 to 0.60	<0.001	0.42	0.30 to 0.58	<0.001	0.36	0.22 to 0.60	<0.001
HLA-B27 negativity	0.37	0.27 to 0.52	<0.001	0.43	0.26 to 0.71	<0.001	0.37	0.26 to 0.54	<0.001	0.37	0.21 to 0.66	<0.001
Education vocational versus compulsory	1.84	1.10 to 3.08	0.02	1.99	0.98 to 4.04	0.056	1.77	1.07 to 2.94	0.03	1.77	0.86 to 3.63	0.12
Education academic versus compulsory	2.64	1.52 to 4.57	<0.001	2.53	1.22 to 5.25	0.01	2.30	1.27 to 4.14	0.01	2.78	1.29 to 5.95	0.01
Body mass index	0.96	0.93 to 1.00	0.03	0.92	0.87 to 0.97	0.001	0.96	0.92 to 1.00	0.04	0.93	0.88 to 0.98	0.01
Current smoking	0.85	0.62 to 1.18	0.34	0.62	0.41 to 0.93	0.02	0.84	0.60 to 1.19	0.34	0.81	0.52 to 1.28	0.37
ASDAS	0.77	0.64 to 0.93	0.01	1.85	1.25 to 2.73	0.002	2.97	2.39 to 3.69	<0.001	6.26	4.56 to 8.59	<0.001
Sacroiliitis on MRI (inflammation)	1.24	0.89 to 1.72	0.21	1.33	0.90 to 1.97	0.15	1.12	0.78 to 1.61	0.55	1.25	0.78 to 2.00	0.35

Response rates in patients with available outcome at 1 year (±6 months), patients having discontinued the biologic in the meantime for reasons other than remission being considered non-responders.

ASDAS, Axial Spondyloarthritis Disease Activity Score; ASDAS-CII, Clinically important improvement in ASDAS; ASDAS-MI, Major improvement in ASDAS; axSpA, axial spondyloarthritis; HLA-B27, human leucocyte antigen-B27; TNFi, tumour necrosis factor inhibitor.

With regard to the CRP response, the relative difference in CRP at 1 year (±6 months), adjusted for baseline CRP, was comparable between very early and established axSpA and between early and established axSpA (online supplemental table S1).

### TNFi discontinuation analyses

The median maintenance duration of the first TNFi was comparable across the three groups: 2.3 years (95% CI 1.5 to 2.9) in very early axSpA, 1.6 years (95% CI 0.9 to 3.2) in early axSpA and 2.3 years (95% CI 2.1 to 2.8) in established axSpA (log-rank test p=0.40; online supplemental figure S3). The reasons for TNFi discontinuation (adverse events, remission, patient’s preference, pregnancy, surgery and other factors) were similarly distributed across groups (p=0.96). The estimated unadjusted hazards of discontinuing the first TNFi were also comparable: 1.08, 95% CI 0.87 to 1.34 for very early versus established axSpA and 1.19, 95% CI 0.90 to 1.56 for early versus established axSpA (table 6).

**Table 6:** Multiple adjusted Cox proportional hazards model for analysis of drug discontinuation of a first TNF inhibitor in very early and early versus established axSpA

Variable	Unadjusted analysis			Adjusted model 1			Adjusted model 2		
	HR	95% CI	P value	HR	95% CI	P value	HR	95% CI	P value
Very early versus established disease	1.08	0.87 to 1.34	0.47	1.00	0.80 to 1.25	0.99	1.05	0.84 to 1.31	0.68
Early versus established disease	1.19	0.90 to 1.56	0.22	1.14	0.87 to 1.51	0.34	1.14	0.86 to 1.51	0.36
Age				1.00	0.99 to 1.00	0.59	1.00	0.99 to 1.00	0.57
Female sex				1.55	1.33 to 1.80	<0.001	1.60	1.37 to 1.86	<0.001
HLA-B27 negativity				1.48	1.26 to 1.75	<0.001	1.48	1.26 to 1.75	<0.001
Education vocational versus compulsory				0.97	0.78 to 1.20	0.76	0.94	0.75 to 1.18	0.59
Education academic versus compulsory				0.88	0.69 to 1.12	0.30	0.86	0.67 to 1.12	0.26
Body mass index							1.01	0.99 to 1.03	0.31
Current smoking							1.16	0.98 to 1.38	0.09
ASDAS							0.91	0.82 to 1.00	0.045
Sacroiliitis on MRI (inflammation)							0.92	0.78 to 1.08	0.31

ASDAS, Axial Spondyloarthritis Disease Activity Score; axSpA, axial spondyloarthritis; HLA-B27, human leucocyte antigen-B27; TNF, tumour necrosis factor.

No significant difference in drug discontinuation rates was observed between very early and established axSpA, or between early and established axSpA, in the adjusted analyses after accounting for potential confounders and baseline disease activity (adjusted models 1 and 2 in table 6). Additional adjustment for the time-period of treatment initiation (2004–2014 vs 2015–2025) had no impact on the hazard of TNFi discontinuation in very early versus established axSpA and in early versus established axSpA (online supplemental table S3). A comparison between the analysis using multiple imputation of missing data in model 2 and a complete case analysis is shown in the online supplemental figure S4. Female sex and HLA-B27 negativity were associated with a significantly higher risk of TNFi discontinuation, whereas a higher ASDAS at baseline was associated with a significantly lower risk (table 6).

## Discussion

Recent studies in axSpA, including a meta-analysis of 11 randomised controlled trials of bDMARDs, have not demonstrated an effect of symptom duration—particularly when early disease is defined as ≤2 years of axial symptoms—on short-term treatment outcomes.^5^,^8^ The choice of a 2-year threshold in the ASAS consensus^4^ was not based on specific research evidence; rather, it was defined by consensus, among expert opinion and it was influenced by the persistently long diagnostic delay in axSpA.^5^ It also aligned with the initial concept of a 2-year therapeutic window of opportunity in rheumatoid arthritis, during which achieving remission is believed to prevent irreversible structural damage.^16^ Therefore, we investigated whether initiating TNFi within a shorter period after the onset of axial symptoms (a disease stage we refer to as ‘very early axSpA’, to distinguish it from the ASAS definition) might influence treatment effectiveness.

We could not demonstrate that treatment response was different between patients with very early disease and patients with established axSpA (symptom duration of at least 2 years). It remains unclear whether earlier expectations may have been based on inaccurate assumptions. Previous studies suggesting a potential benefit of early treatment may have been affected by methodological shortcomings.^17^,^19^ Although these studies included patients with short symptom duration, active treatment was not directly compared with placebo in both early and established disease populations.^20^ This issue was addressed in the referenced meta-analysis,^9^ which did not confirm a short-term advantage of early treatment.

As there is no specific referral strategy within the SCQM registry, other factors may have contributed to the relatively rapid initiation of a bDMARD within 1 year of the onset of back pain in the SCQM registry. First, patients with very early axSpA who initiate a bDMARD have significantly higher subjective and objective disease activity and are therefore more easily identified. We have partially addressed the ensuing potential bias by indication through adjusting the analyses for the presence of MRI inflammation and for ASDAS at baseline. Second, the ASAS criteria for early axSpA only consider the onset of back pain, not the potential presence of other musculoskeletal or extra-musculoskeletal manifestations at an earlier time point. In a relevant proportion of cases, patients may have already been identified based on other inflammatory symptoms, with the additional onset of back pain triggering the decision to initiate a bDMARD. The IQR of the overall symptom duration in very early axSpA is considerably broader than that of axial symptom duration: 0.6–5 years versus 0.1–0.7 years, respectively. Finally, patients who are eligible for bDMARD treatment are preferentially enrolled in the SCQM registry, as Swiss regulatory authorities require ongoing monitoring of all patients receiving costly biologic therapies under the SCQM framework. Additionally, rheumatologists can offset the costs of biologic drugs used for patients enrolled in the SCQM registry against their overall treatment expenditures.

Our study indicates that, in real-world clinical practice,^21^ rheumatologists appropriately select patients for bDMARD therapy even at a very early disease stage. Since the publication of the ASAS classification criteria in 2009,^22^ the diagnostic delay in axSpA has steadily decreased.^23^ However, the proportion of patients with symptom duration ≤1 year included in the SCQM registry has not increased over the past 20 years. This likely reflects the fact that the proportion of patients with very high disease activity, who are most easily identifiable, has remained constant. It also highlights the challenging balance between achieving an early diagnosis and avoiding misdiagnosis.^24^

Some limitations of our analyses must be acknowledged. In addition to the observational design of our study, potential recall bias regarding the onset of symptoms is inherent to the nature of the research question. Because MRIs are not collected systematically in the SCQM registry, no central reading was available to confirm the presence of inflammatory sacroiliac changes at baseline. Furthermore, MRIs are not recommended for routine use to assess treatment response in real-world clinical practice, and as such, were unavailable for evaluating response using this imaging modality. Finally, and perhaps most importantly, the concept of ‘early disease’ is inherently complex and depends on several factors that are difficult or impossible to measure accurately. This makes it unlikely that our analyses could fully adjust for all relevant confounders. Moreover, although our sample size was larger than in previous studies, the CIs indicate that a substantial difference between the groups is unlikely, but cannot be ruled out.

The current analysis only informs us about short-term clinical outcomes. Whether initiating treatment early and maintaining it affects long-term clinical outcomes remains to be determined. A longitudinal association between early axSpA and lower disease activity over 10 years of follow-up has been reported in the German Spondyloarthritis Inception Cohort (GESPIC).^25^ However, these findings require confirmation, as the distinction between early and established disease should ideally be made at the time of diagnosis or treatment start rather than at the more arbitrary time point of inclusion in an observational cohort. Although GESPIC represents an inception cohort, a proportion of patients were already receiving TNFi at inclusion.

The prospect of a ‘window of opportunity’ in axSpA relates to disease modification, such as reducing the long-term structural damage.^17^ Several observational studies have consistently suggested that TNFi may have the potential to limit the accumulation of osteoproliferative changes in the spine.^26^,^31^ The impact of non-steroidal anti-inflammatory drugs or non-pharmacological interventions, such as physical exercise or lifestyle factors, on radiographic progression remains controversial.^32^,^34^ Demonstrating that early diagnosis can reduce radiographic progression will be critical. Although a recent longitudinal analysis attempted to address this issue, classification of early versus late disease was based on the first available radiograph rather than at diagnosis, limiting meaningful conclusions about disease modification.^35^

In conclusion, our study does not indicate higher TNFi effectiveness in patients who initiate treatment within 1 year of symptom onset compared with those who start therapy later.

## Supplementary material

10.1136/rmdopen-2025-006647 online supplemental appendix 1

## Data Availability

Data may be obtained from a third party and are not publicly available.
