# Supplementary material for: Effectiveness of TNF inhibitors in patients with very early axial spondyloarthritis, defined as duration of ≤1 year of back pain: longitudinal observational data from the SCQM registry
Source: RMD Open. 2026 Mar 5;12(1):e006647. doi: 10.1136/rmdopen-2025-006647 (PMC12970104; doi:10.1136/rmdopen-2025-006647)
Supplement: online supplemental appendix 1 [file rmdopen-12-1-s001.docx]

**Supplementary Appendix**

**Effectiveness of TNF inhibitors in patients with very early axial spondyloarthritis (≤1 year of back pain): longitudinal observational data from the SCQM patient registry**

Mauro Bachmann^1^, Andrea Götschi^2^, Annik Steimer^1^, Jonas Brändli^2^, Kristina Bürki^1^, Michael Andor^3^, Claudia Rodrigues^2^, Simon Grosswiler^4^, Martin Wendiggensen^5,6^, Diego Kyburz^7^, Michael J. Nissen^8^, Burkhard Möller^9^, Sabine Adler^9,10^, Diana Dan^11^, Frauke Förger^12^, Oliver Distler^1^, Sofia Ramiro^13,14^, Raphael Micheroli^1^, Adrian Ciurea^1^

**Table S1.** Log-linear regression model for analysis of changes in CRP over time after initiation of a first TNF inhibitor in very early and early vs. established axSpA.

| **Variable** | **β** | **95% CI** |
| --- | --- | --- |
| Very early vs. established disease | 1.05 | 0.84; 1.31 |
| Early vs. established disease | 1.14 | 0.86; 1.51 |

The model estimates the relative difference in CRP at 1 year between the groups while adjusting for baseline CRP.

**Table S2.** Multiple adjusted response analysis (ASDAS<2.1) at 1 year of treatment with a first TNF inhibitor in very early and early vs. established axSpA (model 2) after additional adjustment for the time-period in which the first TNFi was initiated.

| **Variable** | **OR** | **95% CI** | **P** |
| --- | --- | --- | --- |
| Very early vs. established disease | 1.07 | 0.69; 1.68 | 0.76 |
| Early vs. established disease | 1.00 | 0.57; 1.76 | 0.99 |
| Age | 0.99 | 0.97; 1.00 | 0.03 |
| Female sex | 0.40 | 0.30; 0.54 | <0.001 |
| HLA-B27 negativity | 0.38 | 0.27; 0.53 | <0.001 |
| Education vocational vs. compulsory | 1.87 | 1.14; 3.07 | 0.01 |
| Education academic vs. compulsory | 2.68 | 1.56; 4.59 | <0.001 |
| Body mass index | 0.96 | 0.93; 0.99 | 0.02 |
| Current smoking | 0.86 | 0.62; 1.18 | 0.35 |
| ASDAS | 0.78 | 0.65; 0.94 | 0.007 |
| Sacroiliitis on MRI (inflammation) | 1.25 | 0.92; 1.71 | 0.16 |
| Treatment start 2015-2025 versus  treatment start 2004-2014 | 1.14 | 0.85; 1.53 | 0.37 |

ASDAS = Axial Spondyloarthritis Disease Activity Score; HLA-B27 = human leucocyte antigen-B27.

**Table S3.** Multiple adjusted Cox proportional hazards model for analysis of drug discontinuation of a first TNF inhibitor in very early and early vs. established axSpA (model 2) after additional adjustment for the time-period in which the first TNFi was initiated.

| **Variable** | **HR** | **95% CI** | **P** |
| --- | --- | --- | --- |
| Very early vs. established disease | 1.05 | 0.84; 1.31 | 0.69 |
| Early vs. established disease | 1.14 | 0.86; 1.51 | 0.36 |
| Age | 1.00 | 0.99; 1.00 | 0.57 |
| Female sex | 1.60 | 1.37; 1.86 | <0.001 |
| HLA-B27 negativity | 1.48 | 1.26; 1.75 | <0.001 |
| Education vocational vs. compulsory | 0.94 | 0.75; 1.18 | 0.59 |
| Education academic vs. compulsory | 0.86 | 0.67; 1.12 | 0.27 |
| Body mass index | 1.01 | 0.99; 1.03 | 0.31 |
| Current smoking | 1.16 | 0.98; 1.38 | 0.09 |
| ASDAS | 0.91 | 0.82; 1.00 | 0.046 |
| Sacroiliitis on MRI (inflammation) | 0.92 | 0.78; 1.08 | 0.31 |
| Treatment start 2015-2025 versus  treatment start 2004-2014 | 0.98 | 0.84; 1.15 | 0.81 |

ASDAS = Axial Spondyloarthritis Disease Activity Score; HLA-B27 = human leucocyte antigen-B27.


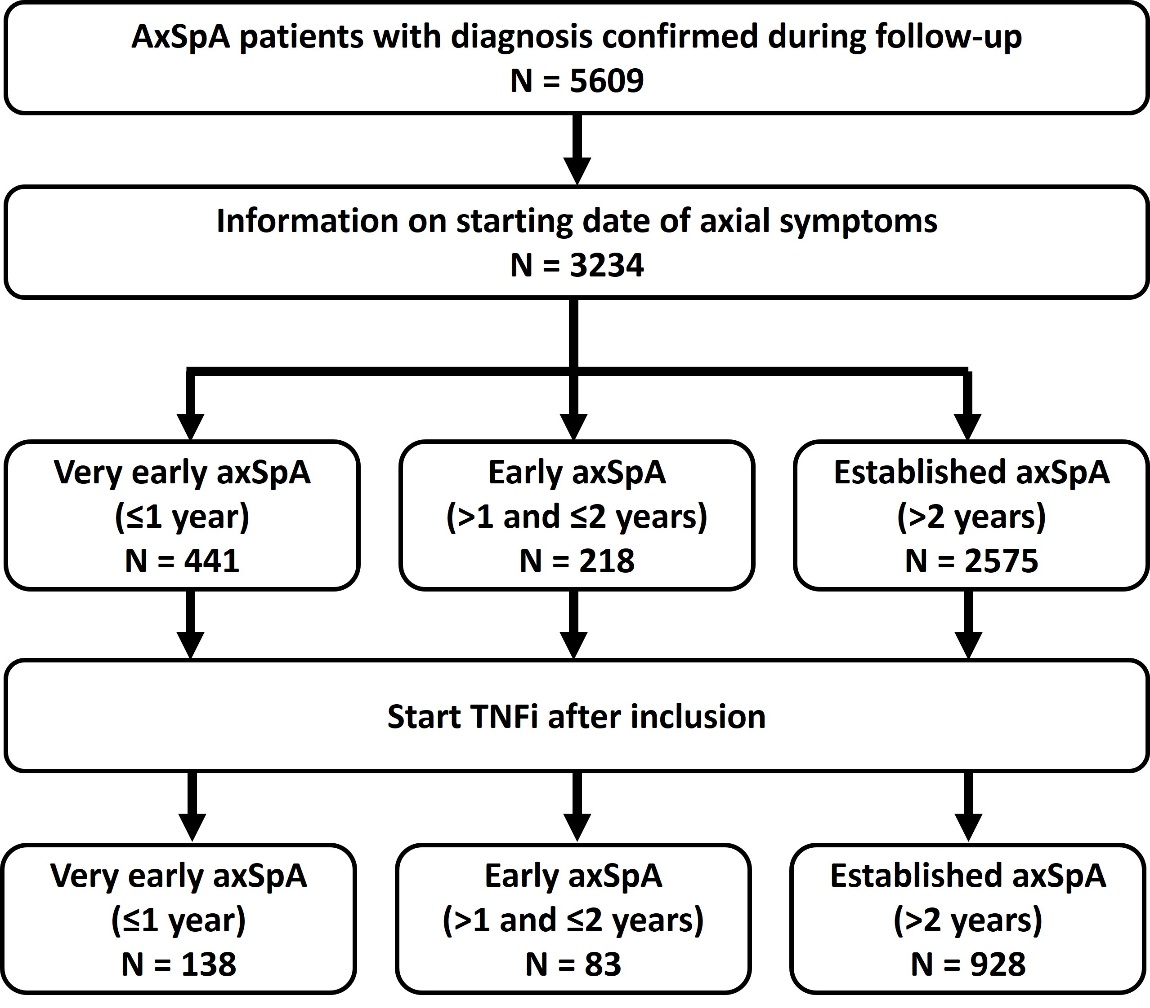


**Figure S1**. Patient disposition in the SCQM cohort regarding axial symptom duration at start of a first TNFi.


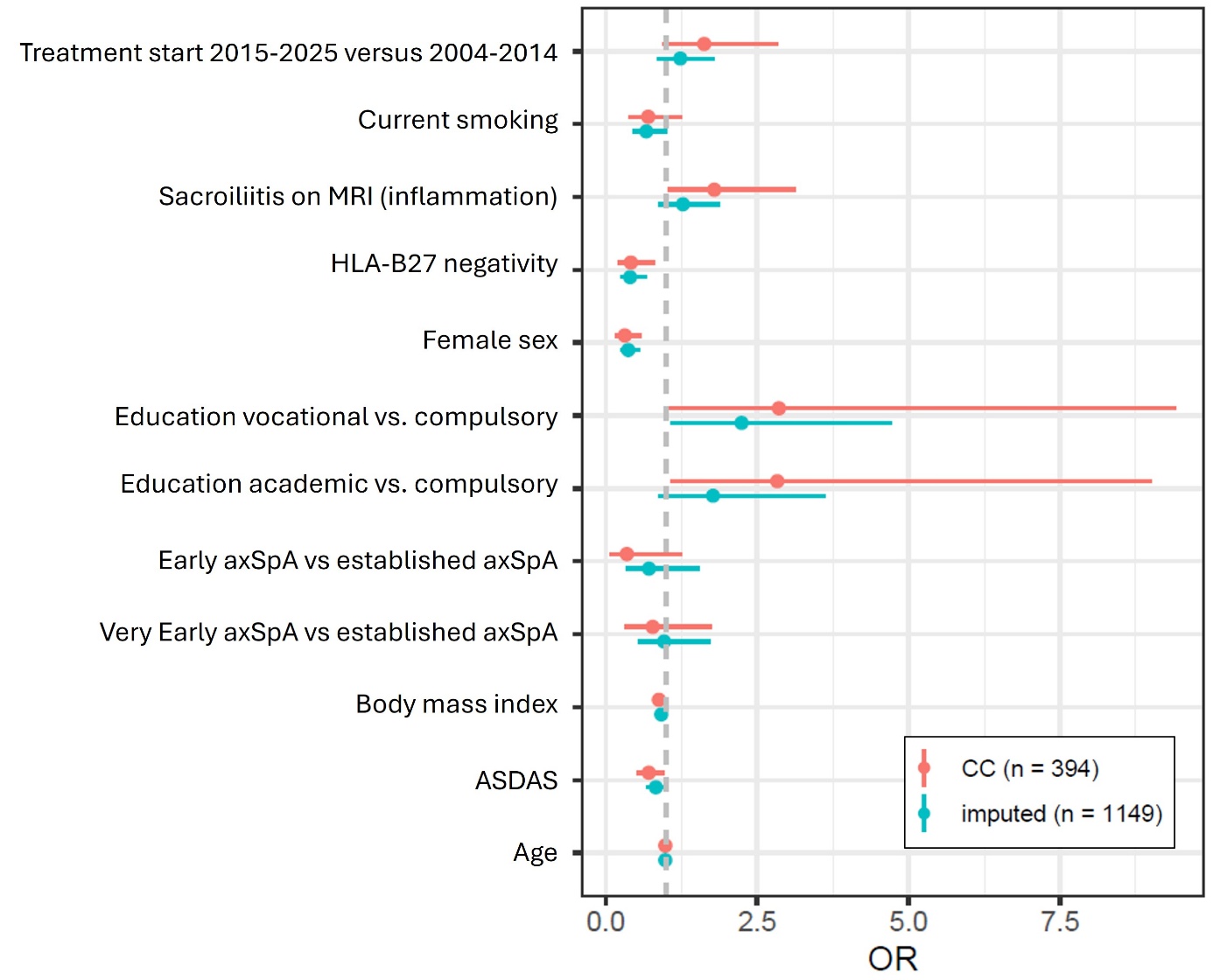


**Figure S2**. Analysis of the achievement of low disease activity (ASDAS<2.1) upon treatment with a first tumour necrosis factor inhibitor in very early vs. established axSpA and early vs. established axSpA. Comparison of the estimated odds ratios and 95% CI in a complete case (CC) analysis in red and in an analysis using multiple imputation by chained equations in green.


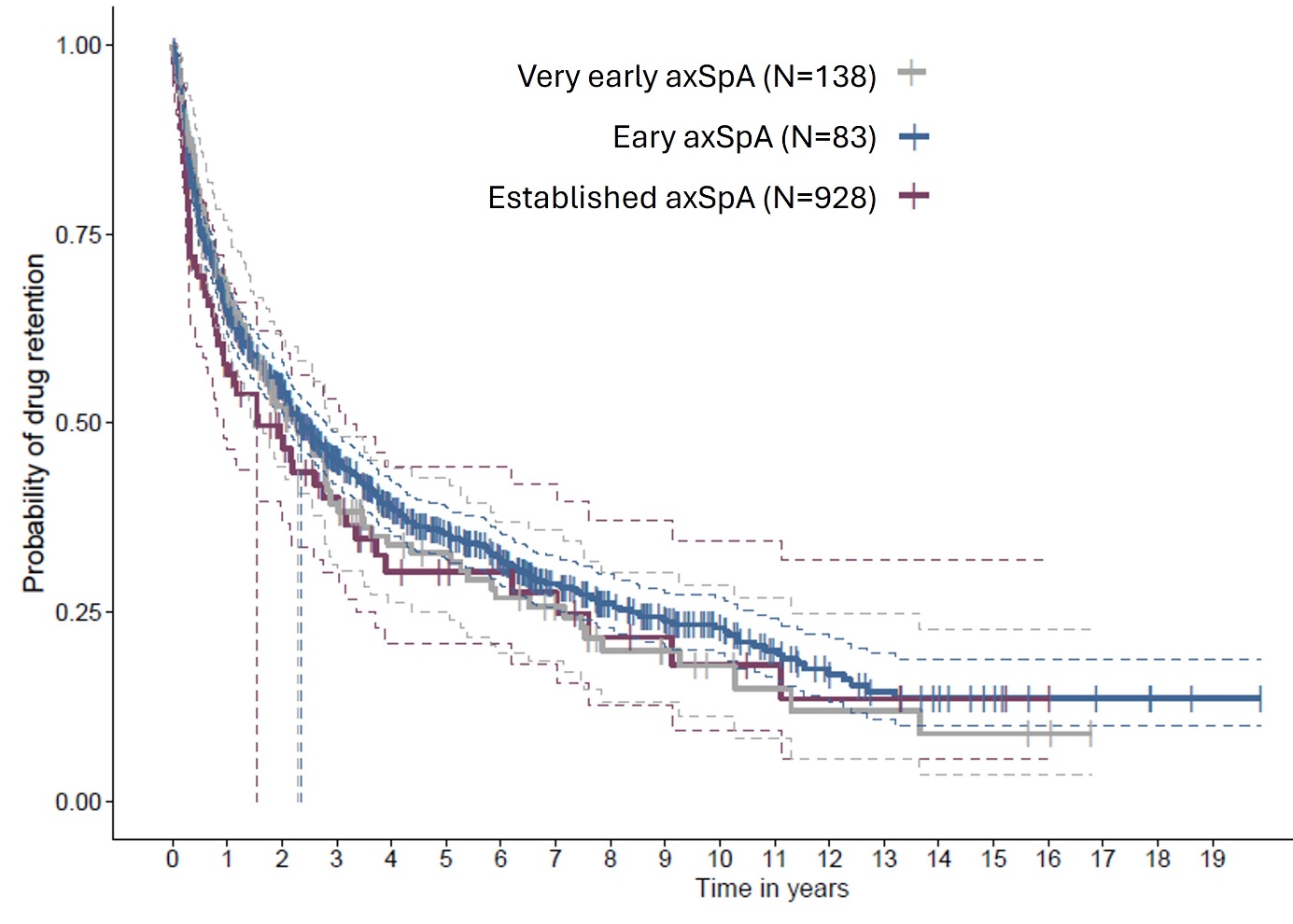


**Figure S3**. Drug survival of the first tumour necrosis factor inhibitor in axial spondyloarthritis (axSpA), stratified by back pain duration at start of treatment: ≤1 year = very early axSpA; >1 and ≤2 years = early axSpA, and >2 years = established axSpA.


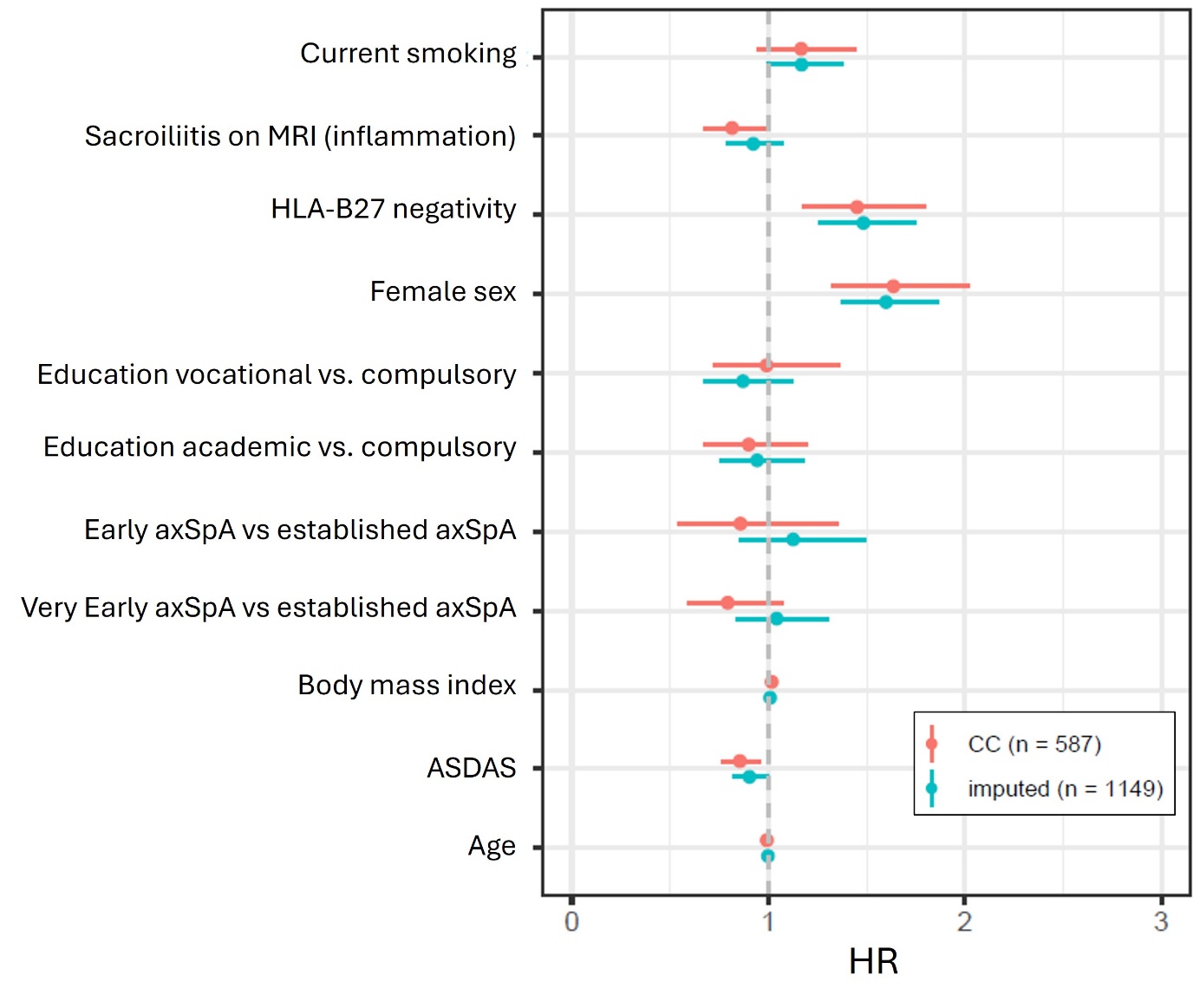


**Figure S4**. Comparison of multiple adjusted cox proportional hazards models for analysis of drug discontinuation of a first tumour necrosis factor inhibitor in very early vs. established axSpA (Hazard radios and 95% CI are shown for the complete case (CC) analysis in red and the analysis using multiple imputation by chained equations in green).
